# Effects of Different Light Qualities on Proliferation and Physiological Characteristics of *Aquilaria sinensis* Tissue-Cultured Seedlings

**DOI:** 10.3390/life15111770

**Published:** 2025-11-19

**Authors:** Le Feng, Chuqi Chen, Chongcheng Yang, Wei Li, Jiapeng Lai, Xiaoyun Min, Siting Gan, Runhua Yi, Chenjun Lin, Feng Feng

**Affiliations:** 1 College of Coastal Agricultural Sciences, Guangdong Ocean University, Zhanjiang 524088, China; fengle@stu.gdou.edu.cn (L.F.); 15014960216@stu.gdou.edu.cn (C.Y.); 17820335211@stu.gdou.edu.cn (W.L.); 11341212hh@stu.gdou.edu.cn (J.L.); scibyrh@gdou.edu.cn (R.Y.); 2 China Forestry Group Leizhou Forestry Bereau Co., Ltd., Zhanjiang 524348, China; chencg@cfgc.cn (C.C.); minxy@cfgc.cn (X.M.); ganst@cfgc.cn (S.G.)

**Keywords:** photobiology, tissue culture, light-emitting diodes, proliferative growth, physiological characterization

## Abstract

In this study, we applied eight different light quality treatments and investigated their effects on the proliferation and physiological characteristics of *Aquilaria sinensis* group-cultivated seedlings in order to screen the best light quality for optimizing group-cultivation fast multiplication technology. The results showed that the highest multiplication rates were obtained with blue light and red light, which were significantly higher than those of white light. Blue light was the most effective in promoting the synthesis of photosynthetic pigments, while red light and blue violet light were favorable for the accumulation of soluble sugars. Correlation analysis showed that the multiplication rate was significantly and positively correlated with plant height, chlorophyll b, total chlorophyll, and soluble sugar content. The comprehensive evaluation indicated that blue light, blue-violet light, and red light was most suitable for fostering proliferation of, and physiological status improvement in, group-cultivated *A. sinensis* seedlings, with their superior performance likely attributable to the combined effects of specific spectral properties and appropriate photosynthetic photon flux density (PPFD) levels. The results of this study provide technical support for light environment regulation for the efficient and rapid propagation of group-cultured *A. sinensis* seedlings.

## 1. Introduction

*Aquilaria sinensis* (Lour.) Spreng. is an evergreen tree of the genus *Aquilaria* of the family Thymelaeaceae [1]. As a precious medicinal aromatic plant unique to China, its resin is widely used in traditional medicine because of its analgesic, sedative, and anti-inflammatory properties [2]. In recent years, with the continuous rise in the market price of agarwood and the fact that natural agarwood requires a long formation cycle and is a scarce resource, large numbers of wild *A. sinensis* trees have been severely damaged due to human logging [3,4]. *A. sinensis* now appears on the list of rare and endangered plants in China, as well as the list of national key protected wild plants [5]. Tissue culture technology, with its advantages of short cycles, high propagation efficiency, and stable genetic traits, is regarded as one of the key approaches for achieving efficient conservation and large-scale propagation of *A. sinensis* [6]. Although preliminary research has been conducted on aspects such as explant disinfection, callus induction, and regeneration system establishment [7,8], practical cultivation of *A. sinensis* tissue-cultured seedlings still commonly faces challenges such as low proliferation rates and inconsistent seedling quality. These issues severely hinder the industrial application of this technology. Therefore, in the context of *A. sinensis* seedlings propagation, the question of how to effectively enhance the proliferation efficiency of tissue-cultured seedlings has become a critical technical challenge that urgently needs to be addressed.

Light quality, as a key environmental factor regulating plant morphogenesis and physiological metabolism, influences photosynthetic traits, material accumulation, and antioxidant defense systems through photoreceptor signaling pathways [9]. Previous studies have demonstrated that different photoperiodic treatments (such as red light versus blue light) can promote shoot differentiation and proliferation in various plant tissue culture seedlings by regulating the synthesis and distribution of soluble sugars and soluble proteins, as well as enhancing the activity of antioxidant enzymes like superoxide dismutase (SOD) and peroxidase (POD) [10,11,12]. Using different light qualities to improve the proliferation efficiency of tissue culture seedlings has achieved certain results in woody plants such as *Aquilaria crassna* [13], *Rhododendron* [14], and *Vaccinium bracteatum* [15]. However, due to the pronounced species specificity in plant responses to light quality, research on how different spectral compositions affect the proliferation of *A. sinensis* tissue culture seedlings and their underlying physiological mechanisms remains largely unexplored.

Therefore, this study aims to systematically investigate the effects of different light quality treatments on the proliferation efficiency and related physiological characteristics of *A. sinensis* tissue culture seedlings. We hypothesize that specific light qualities can effectively promote shoot proliferation and biomass accumulation in *A. sinensis* tissue culture seedlings by regulating chlorophyll synthesis, carbon–nitrogen metabolism, and oxidative homeostasis. To this end, we established eight distinct light quality treatments, focusing on their effects on key indicators, including multiplication rate, chlorophyll content, soluble sugar and protein levels, and antioxidant enzyme activity, in *A. sinensis* tissue-cultured seedlings. This study aims to elucidate the physiological mechanisms underlying light quality regulation of *A. sinensis* tissue-cultured growth, providing theoretical foundations and technical support for optimizing tissue culture light environments and enhancing seedling propagation efficiency.

## 2. Materials and Methods

### 2.1. Experimental Materials

Aseptic seedlings induced from stem segments of 1-year old *A. sinensis* live seedlings and histocultured seedlings with 12 successive proliferation runs were used as test materials. Well-grown proliferating shoots with heights of 4~5 cm were selected and cut into 1 cm high single shoots by retaining the terminal buds, inoculated on proliferation medium (Half-strength Murashige and Skoog (1/2MS) [16] + 0.4 mg/L benzyladenine (BA) + 0.1 mg/L a-naphthalene acetic acid (NAA), 30 g/L sucrose, 6 g/L agar, and potential of hydrogen (pH) 5.8), and cultured under eight different light quality treatments. Each treatment was replicated three times, with 10 bottles inoculated per replication. Each bottle was inoculated with a single shoot segment from a uniformly developed plant, resulting in a total of 30 bottles per treatment.

### 2.2. Different Light Quality Treatments and Culture Conditions

**Figure 1:**
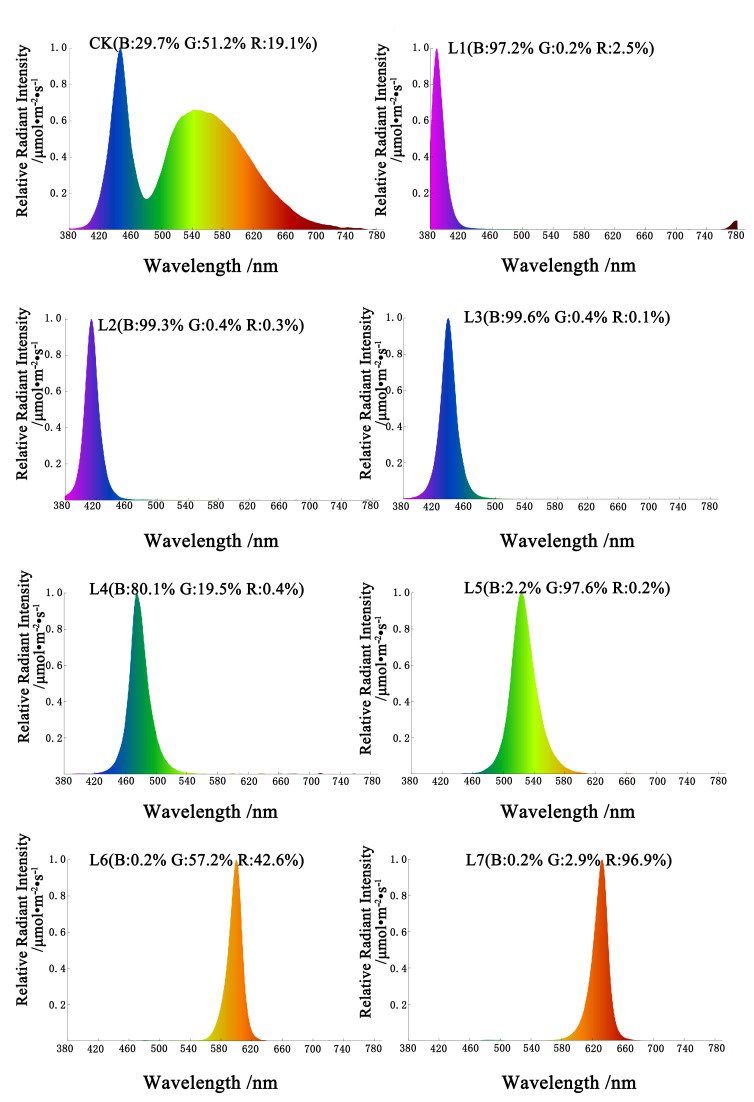
Relative spectral distribution of treatments. CK: white light; L1: purple light; L2: blue-violet light; L3: blue light; L4: blue-green light; L5: green light; L6: orange light; L7: red light. The photosynthetic photon flux density (PPFD) for CK, L1, L2, L3, L4, L5, L6, and L7 was 39.084, 27.451, 85.620, 142.324, 130.849, 73.351, 27.688, and 64.056 µmol m^−2^ s^−1^, respectively.

The tissue-cultured seedlings were subjected to photoperiodic cultivation under eight different light treatments. The spectral properties and photosynthetic photon flux density (PPFD) of the light sources were characterized using a handheld spectroradiometer (HP330P, Hangzhou LCE Intelligent Detection Instrument Co., Ltd., Hangzhou, China). The light source designs are as follows: white light (CK; blue: 29.7%, green: 51.2%, red: 19.1%; 39.084 µmol m^−2^ s^−1^ PPFD), purple light (L1; blue: 97.2%, green: 0.2%, red: 2.5%; 27.451 µmol m^−2^ s^−1^ PPFD), blue-violet light (L2; blue: 99.3%, green: 0.4%, red: 0.3%; 85.620 µmol m^−2^ s^−1^ PPFD), blue light (L3; blue: 99.6%, green: 0.4%, red: 0.1%; 142.324 µmol m^−2^ s^−1^ PPFD), blue-green light (L4; blue: 80.1%, green: 19.5%, red: 0.4%; 130.849 µmol m^−2^ s^−1^ PPFD), green light (L5; blue: 2.2%, green: 97.6%, red: 0.2%; 73.351 µmol m^−2^ s^−1^ PPFD), orange light (L6: blue: 0.2%, green: 57.2%, red: 42.6%; 27.688 µmol m^−2^ s^−1^ PPFD), and red light (L7; blue: 0.2%, green: 2.9%, red: 96.9%; 64.056 µmol m^−2^ s^−1^ PPFD). Relative radiation intensities and wavelengths of the eight light sources are shown in Figure 1. Incubation temperature was always at (25 ± 2) °C, the light time was 12 h/d, and the incubation time was 30 d.

### 2.3. Measurement Indexes and Methods

#### 2.3.1. Determination of Growth Indexes

The multiplication rate and plant height were evaluated as growth indexes. The multiplication rate was defined as the number of single buds obtained from each mother bud after 30 days of cultivation and was calculated as follows: multiplication rate = (number of buds proliferated in 30 days from each mother bud)/(number of inoculated buds). Plant height was measured as the length from the base of the plant to the terminal bud using a straightedge [13].

#### 2.3.2. Methods of Measurement of Physiological Indicators

##### Determination of Pigment Content

The chlorophyll content was determined using the ethanol–acetone mixed extraction method [17]. Briefly, freshly proliferated leaf and branch samples were rinsed with deionized water, blotted dry, and sliced into 2 mm thick pieces. The sliced tissues were thoroughly mixed, and triplicate aliquots were dispensed into 15 mL test tubes. Subsequently, 10 mL of a 95% ethanol–acetone mixed extract (1:1, *v*/*v*) was added to each tube. The tubes were kept in darkness for 48 h to allow for complete extraction and color development. The mixed extract was used as a blank control to calibrate the UV-visible spectrophotometer (UV-5500PC, Shanghai Meitian Instrument Co., Ltd., Shanghai, China) to 100% light transmission. The absorbance of the supernatant was then measured at 663, 645, and 470 nm. The pigment contents were calculated according to the following equations:

Chlorophyll a content (mg/g^−1^): *c*(Ch1a) = (12.7A_663_ − 2.69A_645_) × V/(1000 × W)

Chlorophyll b content (mg/g^−1^): *c*(Ch1b) = (22.9A_645_ − 2.69A_663_) × V/(1000 × W)

Carotenoids (mg/g): *c*(Car) = 1000 × A_470_ − 2.05 × c(Ch1a) − 114.8c(Ch1b)/245

Total chlorophyll content C = *c*(Ch1a) + *c*(Ch1b)

In these equations, V represents the volume of the extracted liquid (mL); W is the fresh weight of the sample (g); and A_663_, A_645_, and A_470_ are the absorbance values at wavelengths of 649, 470, and 665 nm, respectively.

##### Determination of Soluble Sugar Content (SS)

The soluble sugar content was determined by the anthrone colorimetric method [18]. For extraction, a fresh sample (0.5 g) was weighed and transferred into a large test tube. Then, 15 mL of distilled water was added, and the mixture was boiled in a water bath for 20 min. After cooling, the extract was filtered into a 100 mL volumetric flask. The residue was rinsed several times with distilled water, and the volume was made up to the mark. For the assay, 1.0 mL of the extract was mixed with 5 mL of anthrone reagent. The mixture was heated to develop color, and its optical density was measured. This procedure was performed in triplicate. A blank was prepared using 1.0 mL of distilled water mixed with 5 mL of anthrone reagent. The soluble sugar content was calculated using the following formula:

Soluble sugar content (mg/g) = [C × (V/A) × n]/(W × 10^3^)

where C is the sugar (μg) from the standard equation, A is the volume of sample liquid aspirated (mL), V is the total volume of extracted liquid (mL), n is the dilution factor, and W is the sample weight (g).

##### Determination of Soluble Protein (SP) Content

Soluble protein content was determined using the Coomassie Brilliant Blue G-250 method [18]. Fresh leaf samples (0.2 g) were homogenized in a pre-cooled mortar with 5 mL of phosphate buffer (pH 7.4) in an ice bath. The homogenate was centrifuged at 4000 r/min for 10 min. Subsequently, 1 mL of the supernatant was transferred to a test tube, mixed with 5 mL of Coomassie Brilliant Blue G-250 reagent, and vortexed thoroughly. After standing for 5 min, the absorbance of the reaction mixture was measured at 595 nm. The soluble protein content was calculated using the following formula:

Protein content (mg/g) = (C × VT)/(W × Vs × 1000)

where VT is the volume of extract/mL, Vs is the volume of enzyme liquid used in the assay/mL, W is the fresh weight of the sample/g, and C is the SP content of the assay solution obtained by checking the standard curve.

##### Determination of Free Proline (Pro) Content

The free proline content was assayed according to the method of Bates et al. [19]. Fresh leaf samples (0.2 g) from each treatment were weighed, cut into pieces, and placed in test tubes. Then, 5 mL of 3% sulfosalicylic acid solution was added to each tube. The tubes were capped and heated in a boiling water bath for 10 min, with occasional shaking. After cooling to room temperature, 2 mL of the supernatant was pipetted into a new test tube. Then, 2 mL of glacial acetic acid and 2 mL of acid ninhydrin reagent were added, and the mixture was heated in a boiling water bath for 40 min. After cooling, 5 mL of toluene was added to each tube, and the mixture was shaken vigorously for 30 s. The mixture was then allowed to separate, and the upper toluene layer containing the chromophore was collected. Toluene was used as a blank, and the absorbance of the solution was measured at 520 nm. The proline content was calculated as follows:

Free proline (Pro) = [C × (V/A)]/W

where C is the content of proline in the extract (μg/mL), V is the total volume of the extract (mL), A is the volume aspirated in the assay, and W is the sample mass (g)

##### Determination of Malondialdehyde (MDA) Content

The malondialdehyde (MDA) content was determined by the thiobarbituric acid (TBA) method. Fresh leaf samples (0.1 g) were homogenized in 5 mL of 10% trichloroacetic acid (TCA). The homogenate was centrifuged at 4000 r/min for 10 min at room temperature. Then, 2 mL of the supernatant was aliquoted into a test tube, mixed with 2 mL of 0.6% TBA solution, and heated in a boiling water bath for 15 min. The tube was rapidly cooled to room temperature and centrifuged again at 4000 r/min for 10 min. The absorbance of the resulting supernatant was measured at 450, 532, and 600 nm. A control was prepared using 2 mL of 10% TCA mixed with 2 mL of 0.6% TBA. The MDA content was calculated using the following equations:

C_MDA_ = 6.452 × (A_532_ − A_600_) − 0.559 × A_450_

Malondialdehyde content = (C_MDA_ × V_t_)/(V_s_ × W)

where C_MDA_ is the content of MDA (μmol/mL), V_t_ is the overall volume of extract (mL), V_s_ is the volume of extract taken for the determination (mL), and W is the weight of the sample (g)

##### Determination of Different Enzyme Activities

For the extraction of crude enzymes, leaf samples (0.5 g) from each treatment were weighed and homogenized in a pre-cooled mortar with 5 mL of pre-cooled 0.05 mol/L phosphate buffer (pH 7.8). The homogenate was transferred to a volumetric flask, and the final volume was adjusted to 10 mL with the same buffer. The solution was shaken thoroughly and then centrifuged at 8000 r/min for 20 min at 4 °C. The resulting supernatant was collected and used for the assays of superoxide dismutase (SOD), peroxidase (POD), and catalase (CAT) activities. The procedures for these enzyme activity assays were performed as described in the literature [20,21,22].

### 2.4. Data Analysis

The test results were statistically analyzed and graphed using Excel 2010, SPSS 25.0, and GraphPad Prism 10 software, and the significance of the differences in each index between treatments was tested using one-way ANOVA (One-way ANOVA) and the least significant difference method (SSR) [23]. Pearson’s method was used for a two-tailed test of correlation among morphological indexes, leaf anatomical structure indexes, and chlorophyll and photosynthetic indexes. The drought tolerance values of the six species of Jackfruit were calculated and ranked by principal component analysis, the subordinate function method and the principal component weighting method.

Firstly, the data of each index were standardized and subjected to principal component analysis, and the principal components were extracted according to the principle that the characteristic root was greater than 1 and the cumulative contribution rate was greater than 85%, and the loadings of each principal component were calculated according to Formula (1), and the score value of the principal component of each tree species was calculated by constructing the linear expression between each principal component and each index (C). The affiliation function value (U) of the principal component score value of each tree species was calculated according to Formula (2). According to the size of the contribution rate of the principal components, Formula (3) was used to calculate the corresponding weights (*W_i_*), and the comprehensive evaluation value of drought resistance of each species (*D*) was calculated according to Formula (4).

(1) $$t_{i}=a_{i}/\sqrt{\lambda i}\left(\mathrm{i}=1,2,\ldots ,|n\right)$$

where $t_{i}$ is the coefficient of the *i*th principal component factor, $a_{i}$ is the loading vector of the *i*th principal component factor, and λi is the eigenroot of the *i*th principal component factor.

(2) $$U_{i}=\left(X_{i}-X_{min}\right)/\left(X_{\max}-X_{min}\right)$$

where *U_i_* is the value of the affiliation function of the score value of the *i*th principal component, *X_i_* is the score value of the *i*th principal component, *X_min_* is the minimum value of the score value of the *i*th principal component, and *X_max_* is the maximum value of the score value of the *i*th principal component.

(3) $$W_{i}=P_{i}/\sum_{i=1}^{n}P_{i}\left(i=1,2,\ldots ,n\right)$$

(4) $$D=\sum_{i=1}^{n}\left(U_{i}\times W_{i}\right)$$

where *W_i_* is the weight of the *i*th composite assessment value among all composite assessment values, *P_i_* is the contribution rate of the ith composite assessment value, and *D* is the composite assessment value.

## 3. Results

### 3.1. The Effect of Different Light Treatments on the Proliferation and Growth of A. sinensis Seedlings

**Figure 2:**
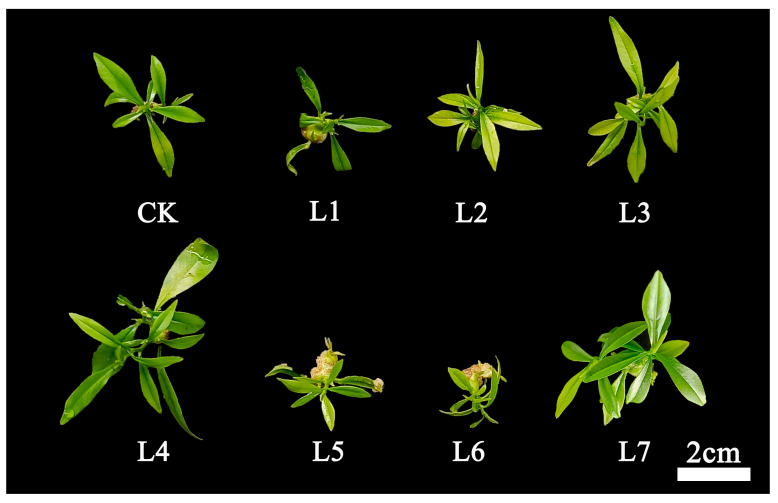
Effect of different light treatments on the proliferation and growth of *Aquilaria sinensis* tissue-cultured seedlings. CK: white light; L1: purple light; L2: blue-violet light; L3: blue light; L4: blue-green light; L5: green light; L6: orange light; L7: red light. The photosynthetic photon flux density (PPFD) for CK, L1, L2, L3, L4, L5, L6, and L7 was 39.084, 27.451, 85.620, 142.324, 130.849, 73.351, 27.688, and 64.056 µmol m^−2^ s^−1^, respectively.

The effect of different light treatments on the proliferation and growth of *A. sinensis* seedlings is shown in Figure 2. It can be found that *A. sinensis* seedlings treated with white light (CK), blue light (L3), blue-green light (L4), and red light (L7) can proliferate and grow normally, with light green or dense green leaves and less basal healing tissue. Application of purple light (L1), green light (L5) and orange light (L6) yielded more basal healing tissues, while the blue-violet light (L2) yielded yellowish leaves, meaning this light quality was unfavorable for the proliferation and growth of *A. sinensis*.

In terms of multiplication rate, significant differences were observed among the different light quality treatments (Table 1). The blue-green light (L4) and red light (L7) treatments exhibited the highest growth coefficients, significantly higher than the other treatments, indicating that these two light qualities effectively promote tissue culture plant proliferation. Blue light (L3) showed the next highest growth coefficient, significantly higher than the control. Conversely, the purple light (L1), blue-violet light (L2), blue-green light (L4), and orange light (L6) treatments exhibited lower growth coefficients, significantly lower than those of blue light (L3), blue-green light (L4), and red light (L7). This indicates that blue-green light (L4) and red light (L7) are optimal for inducing cell division and shoot proliferation. Regarding plant height, the red light (L7) treatment yielded the tallest plants, showing no significant difference from blue-green light (L4) but significantly exceeding other treatments. The blue light (L3) and CK treatments exhibited similar plant heights, with both being at relatively high levels. Plants under purple light (L1), blue-violet light (L2), blue-green light (L4), and orange light (L6) exhibited shorter heights, indicating these light treatments inhibited seedling elongation growth. Observations of shoot growth aligned with these data. The blue-green light (L4), red light (L7), and blue light (L3) treatments displayed pale green leaves with vigorous growth and minimal callus tissue at the base, suggesting that these light qualities promoted healthy shoot formation and development. Conversely, purple light (L1), blue-green light (L4), and orange light (L6) showed poor growth with extensive callus formation at the base, suggesting that these light treatments may have induced undesirable dedifferentiation and callus formation, hindering normal bud development. Blue-violet light (L2) exhibited pale yellow leaves and poor growth, potentially indicating light inhibition or nutritional metabolism issues.

### 3.2. Effects of Different Light Qualities on Photosynthetic Pigment Content of A. sinensis Histocultured Seedlings

**Figure 3:**
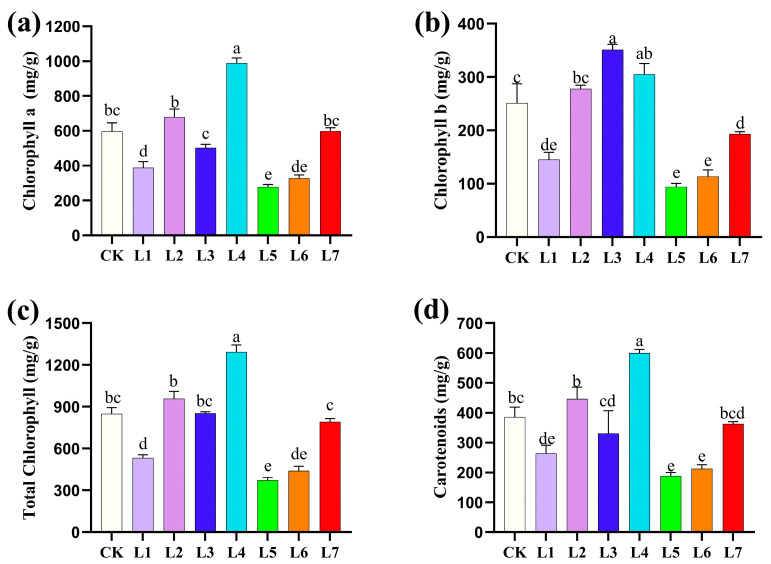
Effect of different light quality treatments on photosynthetic pigment content of *Aquilaria sinensis* tissue-cultured seedlings. The error bars in the figure represent standard errors. Different lowercase letters indicate significant differences between treatments (*p* < 0.05). The photosynthetic photon flux density (PPFD) for CK, L1, L2, L3, L4, L5, L6, and L7 was 39.084, 27.451, 85.620, 142.324, 130.849, 73.351, 27.688, and 64.056 µmol m^−2^ s^−1^, respectively. (**a**): Chlorophyll a content of *A. sinensis*; (**b**): Chlorophyll b content of *A. sinensis*; (**c**): Total Chlorophyll content of *A. sinensis*; (**d**): Carotenoid content of *A. sinensis*.

From the data in Figure 3, it can be seen that there were significant differences in the effects of different LED light quality treatments on the photosynthetic pigment content of *A. sinensis* histocultured seedlings. In terms of chlorophyll a content, blue-green light (L4) treatment had the highest chlorophyll a content, which was significantly higher than all other treatments, followed by blue-violet light (L2) treatment, while the white light (CK) and red light (L7) treatments had similar content (both were at an intermediate level). The chlorophyll a content values for the purple light (L1), blue light (L3), green light (L5), and orange light (L6) treatments were relatively low, with green light (L5) yielding the lowest content. This indicates that blue-green light (L4) is most favorable for chlorophyll a synthesis. In terms of chlorophyll b content, the blue light (L3) treatment had the highest content, followed by the blue-green light (L4) treatment, both of which were significantly higher than the control, the blue-violet light (L2) treatment was at the same level of significance as white light (CK). The chlorophyll b content was lower with the purple light (L1), green light (L5), orange light (L6), and red light (L7) treatments, with the green light (L5) and orange light (L6) treatments having the lowest content. It is noteworthy that the red light (L7) treatment had a relatively low chlorophyll b content, although it performed well in other indicators. In terms of total chlorophyll content, the blue-green light (L4) treatment was significantly highest, followed by the blue-violet light (L2) treatment. White light (CK), blue light (L3), and red light (L7) were in the middle range. The purple light (L1), green light (L5), and orange light (L6) treatments had significantly lower total chlorophyll content, with the green light (L5) treatment being the lowest. The trend of total chlorophyll content was more consistent with that regarding chlorophyll a, indicating that chlorophyll a was the main determinant of total chlorophyll content. In terms of carotenoid content, the blue-green light (L4) treatment also performed optimally, followed by the blue-violet light (L2) treatment. The white light (CK) and red light (L7) treatments were in the middle of the range. The carotenoid contents of the purple light (L1), blue light (L3), green light (L5), and orange light (L6) treatments were lower, with that of the green light (L5) treatment being the lowest.

### 3.3. Effect of Different Light Qualities on Soluble Solids Contents of A. sinensis Histocultured Seedlings

**Figure 4:**
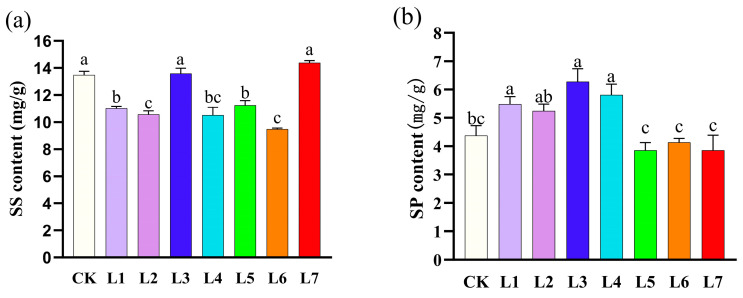
Effect of different light quality treatments on soluble solids contents of *Aquilaria sinensis* tissue-cultured seedlings. (**a**) Soluble sugar content; (**b**) soluble protein content. The error bars in the figure represent standard errors. Different lowercase letters indicate significant differences between treatments (*p* < 0.05). The photosynthetic photon flux density (PPFD) for CK, L1, L2, L3, L4, L5, L6, and L7 was 39.084, 27.451, 85.620, 142.324, 130.849, 73.351, 27.688, and 64.056 µmol m^−2^ s^−1^, respectively.

As can be seen from Figure 4a, the soluble sugar content of *A. sinensis* was significantly different under different light quality conditions. The red light (L7) treatment yielded the highest soluble sugar content, followed by blue light (L3) and white light (CK), with there being no significant differences among the three. This indicated that red light (L7) and blue light (L3) were comparable, or superior, to white light (CK) in promoting soluble sugar accumulation. In contrast, the orange light (L6) treatment had the lowest soluble sugar content by a significant margin, indicating that this light quality condition severely inhibited carbohydrate synthesis or accumulation. The soluble sugar content for the purple light (L1), blue-violet light (L2), blue-green light (L4), and green light (L5) treatments was at a moderately low level. No significant differences were observed among these treatments, but all were significantly lower than those in the white light (CK), blue light (L3), and red light (L7) treatments. This comprehensive analysis showed that red light (L7) and blue light (L3) were the best light qualities for promoting soluble sugar accumulation, while orange light (L6) had an obvious inhibitory effect.

As shown in Figure 4b, the soluble protein contents of *A. sinensis* plants were significantly different under different light quality conditions. The blue light (L3) treatment had the highest soluble protein content (6.27 mg/g) and had the highest level of significance. The purple light (L1) and blue-green light (L4) treatments also had higher contents, which were at the same level of significance as blue light (L3), indicating that these three light qualities were the most favorable for the synthesis and accumulation of soluble proteins. The soluble protein content of the green light (L5), orange light (L6), and red light (L7) treatments were significantly lowest, respectively. The content of the white light (CK) was at a moderately low level. This comprehensive analysis indicated that blue light (L3), purple light (L1), and blue-green light (L4) were the dominant light qualities that promoted soluble protein accumulation.

### 3.4. Effect of Different Light Qualities on Proline and Malondialdehyde in A. sinensis Histocultured Seedlings

**Figure 5:**
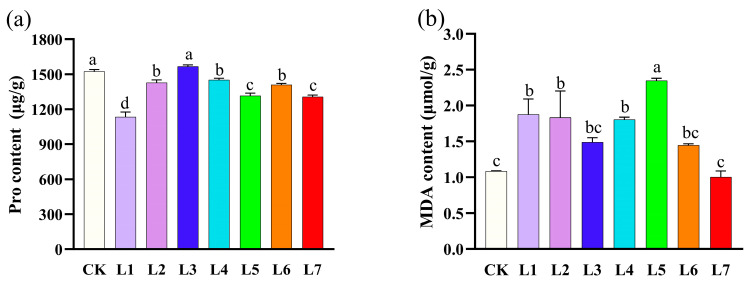
Effect of different light quality treatments on proline and malondialdehyde content of *Aquilaria sinensis* tissue-cultured seedlings. (**a**) Proline content; (**b**) malondialdehyde content. The error bars in the figure represent standard errors. Different lowercase letters indicate significant differences between treatments (*p* < 0.05). The photosynthetic photon flux density (PPFD) for CK, L1, L2, L3, L4, L5, L6, and L7 was 39.084, 27.451, 85.620, 142.324, 130.849, 73.351, 27.688, and 64.056 µmol m^−2^ s^−1^, respectively.

As can be seen in Figure 5a, different LED light quality treatments showed significant differences in proline content accumulation in histocultured seedlings. The proline content in the blue light (L3) and white light (CK) treatment groups was significantly higher than in the other treatment groups. The blue-violet light (L2), blue-green light (L4), and orange light (L6) treatments yielded intermediate levels of proline content, while the purple light (L1) treatment had the lowest proline content. The comprehensive analysis showed that blue light (L3) and white light (CK) were the best light quality conditions for promoting proline accumulation.

As shown in Figure 5b, different LED light quality treatments showed significant differences in malondialdehyde (MDA) accumulation in histocultured seedlings. Green light (L5) treatment had the highest MDA content, significantly higher than all other treatments. The purple light (L1), blue-violet light (L2), and blue-green light (L4) treatments also showed high levels of MDA content. In contrast, the white light (CK) and red light (L7) treatments had the lowest MDA content, and the mean value for the red light (L7) treatment was slightly lower than that of white light (CK).

### 3.5. Effect of Different Light Qualities on Enzyme Activities of A. sinensis Histocultured Seedlings

**Figure 6:**
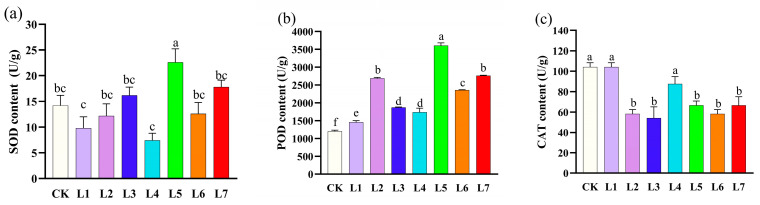
Effect of different light quality treatments on enzyme activity of *Aquilaria sinensis* tissue-cultured seedlings. The error bars in the figure represent standard errors. Different lowercase letters indicate significant differences between treatments (*p* < 0.05). The photosynthetic photon flux density (PPFD) for CK, L1, L2, L3, L4, L5, L6, and L7 was 39.084, 27.451, 85.620, 142.324, 130.849, 73.351, 27.688, and 64.056 µmol m^−2^ s^−1^, respectively. (**a**) SOD content; (**b**) POD content; (**c**) CAT content.

As can be seen from Figure 6a, the effects of different LED light quality treatments on the superoxide dismutase (SOD) activity of histocultured seedlings were significantly different. The green light (L5) treatment had the highest SOD activity, significantly higher than all other treatments. The red light (L7) and blue light (L3) treatments had higher SOD activities, but they resembled the same levels as the white light (CK), blue-violet light (L2), and orange light (L6) treatments, and these values were not significantly different from each other. In contrast, the purple light (L1) and blue-green light (L4) treatments had the lowest SOD activity, significantly lower than the remaining treatments. The combined analysis indicated that green light (L5) induced a significant increase in SOD activity.

As shown in Figure 6b, the effects of different LED light quality treatments on the peroxidase (POD) activity of histocultured seedlings showed significant differences. The POD activity of the green light (L5) treatment was significantly higher than that of all of the other treatments, and its activity was about three times higher than that of white light (CK). The POD activities of the blue-violet light (L2) and red light (L7) treatments were at a similar same level, and both of them had similar activities. The activity of the purple light (L1) treatment was higher than that of the white light (CK) treatment, which had the lowest level of POD activity among all the treatments.

The effects of different LED light quality treatments on the catalase (CAT) activity of histocultured seedlings showed significant differences, as shown in Figure 6c. The highest CAT activities were achieved with the white light (CK), purple light (L1), and blue-green light (L4) treatments, which were significantly higher than the other levels. Red light (L7), green light (L5), blue-violet light (L2), blue light (L3), and orange light (L6) did not differ significantly from each other.

### 3.6. Correlation Analysis of Different Light Qualities on A. sinensis Histocultured Seedlings

**Figure 7:**
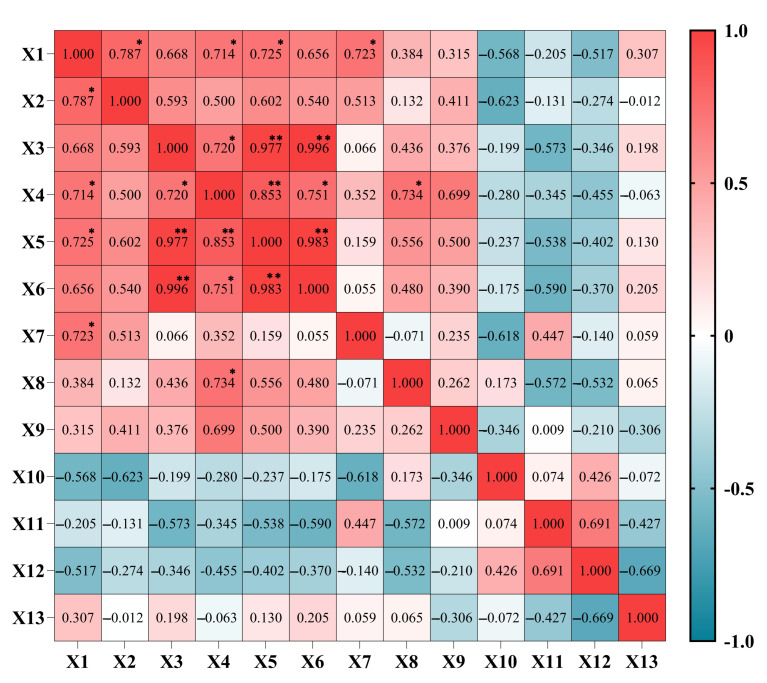
Correlation analysis of different light quality treatments on *Aquilaria sinensis* tissue-cultured seedlings. X1: Value-added coefficient; X2: plant height; X3: chlorophyll a; X4: chlorophyll b; X5: total chlorophyll; X6: carotenoids; X7: soluble sugar; X8: soluble protein; X9: proline; X10: malondialdehyde; X11: superoxide dismutase (SOD); X12: peroxidase (POD); X13: catalase (CAT). * indicates significant correlation (*p* < 0.05); ** indicates highly significant correlation (*p* < 0.01).

The multiplication rate and plant height of *A. sinensis* seedlings grown under eight light quality treatments were correlated, and the results are shown in Figure 7. The multiplication rate was significantly and positively correlated with plant height, chlorophyll b, and total chlorophyll and soluble sugar content. Chlorophyll a was significantly and positively correlated with chlorophyll b and highly significantly and positively correlated with total chlorophyll and soluble sugar. Chlorophyll b was highly and positively correlated with total chlorophyll and significantly correlated with carotenoids and soluble proteins. Total chlorophyll was highly and positively correlated with carotenoids.

Principal component analysis was performed on 13 indicators of *A. sinensis* histocultured seedlings under eight light quality treatments, and the results are shown in Table 2. According to the principle that the characteristic root was greater than one and the cumulative contribution rate was greater than 85%, four principal components were extracted, which were represented by C1, C2, C3, and C4. The cumulative contribution rate reaches 88.338%, indicating that these four principal components can represent the main information of the original indexes and convert the original 13 individual indexes into four comprehensive indexes that are independent of each other. The higher principal component loadings of multiplication rate, chlorophyll a, chlorophyll b, total chlorophyll, and carotenoids for the first principal component indicated that the first principal component was mainly determined by these four factors; the higher principal component loadings of plant height, soluble sugar, superoxide dismutase, and malondialdehyde for the second principal component indicated that the second principal component was mainly determined by these four factors. The principal component loadings for peroxidase, proline, and malondialdehyde are relatively high, indicating that the third principal component is primarily determined by these four factors. The fourth principal component had higher soluble protein and proline principal component loadings, indicating that the fourth principal component was mainly determined by these two factors. Based on the principal component loadings in Table 2, the linear expressions between each principal component and each indicator are presented in Supplementary Table S1.

### 3.7. Comprehensive Evaluation of Different Light Quality Adaptations of Group-Cultivated A. sinensis Seedlings

According to the linear expression formula, the scores of each principal component of different light quality adaptations of *A. sinensis* seedlings were calculated, and the comprehensive evaluation values (D values) of different light quality adaptations, obtained by using the affiliation function method and the method of assigning weights to the principal components, are as shown in Table 3. The size of the D value indicated the ability of *A. sinensis* to adapt to different light qualities, and a larger value indicated that the light quality was more suitable for the proliferation and growth of *A. sinensis* seedlings. The ranking of the adaptability of *A. sinensis* to the eight light qualities was blue-green light (L4) > blue light (L3) > red light (L7) > white light (CK) > blue-violet light (L2) > orange light (L6) > green light (L5) > purple light (L1), which indicated that the light treatments of blue-green light (L4), blue light (L3), and red light (L7) were better than that of white light (CK), and purple light (L1) was the worst, according to the available index data.

## 4. Discussion

Light quality affects many aspects of plant morphogenesis, growth, and development, including photosynthetic physiology and metabolic physiology [24]. Photomodulation is an effective environmental control technique in plant tissue culture, which can influence the effects of plant healing induction, differentiation, and proliferation by regulating light quality [25]. The pshotosynthetically active radiation (PAR) required for plant growth is between wavelengths of 400 and 700 nm, with the highest photosynthetic efficiency being in the blue (450 nm) and red (650 nm) light regions [26]. The response to light varies among plant species. Consequently, the effects of specific light quality combinations on the proliferation of healing tissues and somatic cell embryogenesis also differ significantly [27]. Yongmei Miao et al. found that red light treatment promoted the growth and bud differentiation of *Pholidota cantonensis* histocultures, but the pseudobulbs and leaves were elongated, while other monochromatic light treatments inhibited growth and bud differentiation. The pseudobulbs became shorter and thicker, and leaves became shorter and wider under the blue light treatment [28]. Duosi Liao et al. found that pure red light was most suitable for the proliferation of *Rhododendron lapponicum* ‘Bob’s yellow’; pure blue light was suitable for the proliferation of yellow cup azalea; and mixed red, blue, and green light inhibited the proliferation of both alpine azalea species but promoted chlorophyll synthesis [14]. In this study, the effects of different light qualities on the proliferation and growth of group-cultured *A. sinensis* seedlings and a number of physiological indexes were systematically analyzed by using white light (CK) and seven monochromatic lights with different wavelengths, and the adaptive ordering of the light quality treatments was clarified by the method of principal component analysis and comprehensive evaluation. Notably, the photosynthetic photon flux density (PPFD) varied considerably among treatments, measuring 39.084, 27.451, 85.620, 142.324, 130.849, 73.351, 27.688, and 64.056 µmol m^−2^ s^−1^ for CK, L1, L2, L3, L4, L5, L6, and L7, respectively. This variation indicates that light intensity, in addition to spectral composition, differed across the light quality treatments, which may have contributed to the observed physiological responses. In this study, light quality had a significant regulatory effect on the growth and development of group-cultivated *A. sinensis* seedlings, in which blue-green light (L4), blue light (L3), and red light (L7) were outstanding in promoting proliferation and improving physiological status, and these treatments were characterized by relatively high PPFD levels. The purple light (L1), blue-violet light (L2), green light (L5), and orange light (L6) significantly inhibited growth. The poor performance of purple light (L1) and orange light (L6), in particular, might be partially associated with their lower PPFD values, whereas blue-violet light (L2) and green light (L5), despite having moderate PPFD, still resulted in significant growth inhibition, suggesting a stronger inherent inhibitory effect of their specific light spectra. The highest multiplication rates were found with the blue-green light (L4) and red light (L7) treatments, which indicated that these two light treatments had a significant advantage in promoting shoot differentiation and proliferation. The purple light (L1), blue-violet light (L2), green light (L5), and orange light (L6) treatments had significantly lower multiplication rates, and green light (L5) and orange light (L6) were accompanied by more healing tissue formation, suggesting that these light treatments may have induced a poor dedifferentiation process and inhibited the development of normal shoots [29].

The regulation of photosynthetic pigment content by different light treatments may vary depending on plant species and tissue organs, while the mechanism of action of light to promote the synthesis of photosynthetic pigments may be due to the joint action of photosensitizing pigments and cryptochromes [30,31]. In this study, blue-green light (L4) treatment was the most effective in promoting chlorophyll a, total chlorophyll, and carotenoid synthesis in *A. sinensis* histolytica seedlings, which is consistent with findings in plants such as *Aquilaria crassna* [13], *Phalaenopsis aphrodite* [32], and *Vaccinium bracteatum* [15]. The high PPFD of blue-green light (L4) likely provided ample energy for photosynthetic pigment synthesis, potentially acting synergistically with its specific spectral properties. In contrast, both the green light (L5) and orange light (L6) treatments produced significant inhibitory effects on the synthesis of all types of photosynthetic pigments. Regarding orange light (L6), its very low PPFD likely limited the energy available for pigment production, while the inhibition under green light (L5), despite a moderate PPFD, underscores a potentially negative regulatory role of the green light spectrum itself. These results reveal the important roles of different light qualities in regulating the development of photosynthetic apparatus in *A. sinensis* histocultured seedlings and provide a theoretical basis for optimizing the photosynthetic performance of histocultured seedlings through light quality regulation.

Light quality affects carbohydrate and protein metabolism in higher plants, with red light being more favorable for carbohydrate accumulation, while the addition of blue light favors protein and proline formation [33,34]. In this study, red light (L7) and blue light (L3) treatments showed the best performance in terms of soluble sugar accumulation, which was not significantly different from the control, indicating that these two light qualities favored carbohydrate accumulation. The favorable soluble sugar accumulation under blue light (L3) and red light (L7) may be attributed to a combination of their specific light spectra and their substantial PPFD levels, supporting adequate photosynthesis. The orange light (L6) treatment, on the other hand, had the lowest soluble sugar content, which might be related to the low photosynthetic efficiency driven by its very low PPFD. In terms of soluble proteins, the blue light (L3), purple light (L1) and blue-green light (L4) treatments performed optimally, indicating that these light qualities were the dominant light qualities to promote soluble protein accumulation.

When external light conditions are altered, reactive oxygen radicals are generated within the plant, which can trigger peroxidation of membrane lipids, resulting in the production of MDA [35]. However, antioxidant enzymes in plants, such as superoxide dismutase (SOD), peroxidase (POD), and catalase (CAT), are effective in preventing the accumulation of reactive oxygen species and avoiding the over-oxidation of cell membrane lipids, which, in turn, slows down the aging process of plants [36]. In this study, the green light (L5) treatment showed a significant increase in SOD and POD activities, as well as the highest MDA content, suggesting that this light quality may have induced stronger oxidative stress, leading to an increase in membrane lipid peroxidation, and these findings were consistent with those of Gong Huiting et al., who studied green light’s effect on *Polygonum multiflorum* [35]. The antioxidant enzyme defense mechanisms vary across plant species, due to factors such as the plant’s stage of growth and development [35,37]. In this study, the enzyme activities of *A. sinensis* showed a non-significant correlation with multiplication rate and chlorophyll, and further discussion is needed regarding the effect of light quality on *A. sinensis* histocultured seedlings.

The evaluation of the affiliation function can be used as a screening method for high-quality light culture environment for plant seedlings [38]. In this study, the light quality adaptability was ranked as blue-green light (L4) > blue light (L3) > red light (L7) > white light (CK) > blue-violet light (L2) > orange light (L6) > green light (L5) > purple light (L1) by the combined evaluation of principal component analysis and the affiliation function method, and blue-green light (L4) light quality was superior in several key indexes, such as multiplication rate, chlorophyll content, etc., meaning it could be used as the preferred light treatment during the proliferation stage of group-cultivated *A. sinensis* seedlings. The superior performance of L4 likely results from a beneficial combination of its specific blue-green spectrum and a high PPFD. Blue light (L3) and red light (L7) were slightly inferior to blue-green light (L4) in some aspects, but still significantly better than the control, and have potential for practical application. The promising results for blue light (L3) and red light (L7) also correspond to their relatively high PPFD values, suggesting that the optimal light environment for *A. sinensis* proliferation should consider both spectral quality and sufficient light intensity.

This study reveals, for the first time, the effects of different light qualities on the proliferation and physiological characteristics of *A. sinensis* seedlings and provides a theoretical basis for optimizing the light environment of *A. sinensis*. The observed physiological differences are likely influenced by the combined effects of light spectrum and the varying PPFD across treatments. In the future, we can further explore the spectral composition of blue-green light (L4), blue light (L3), and red light (L7) and their interactions with endogenous plant hormones and analyze the molecular basis of light quality in regulating the proliferation of group-cultivated *A. sinensis* seedlings by combination with transcriptomic or proteomic methods. In addition, the synergistic effects of light quality with other culture factors, such as medium composition and hormone ratios, are also worthy of further study.

## 5. Conclusions

Light quality is a key environmental factor affecting the proliferation efficiency of group-cultured *A. sinensis* seedlings. The multiplication rates, plant heights, photosynthetic pigment contents, osmoregulatory substances, and antioxidant enzyme activities of the histocultured seedlings showed significant differences under different light quality treatments. In this study, blue-green light (L4) and red light (L7) could significantly promote the proliferation of *A. sinensis* seedlings, yielding the highest multiplication rate and robust plant growth, as well as ensuring fewer healing tissues at the base, representing the optimal light quality for inducing bud proliferation. The blue-green light (L4) treatment was most capable of promoting the synthesis of photosynthetic pigments, and it could significantly increase chlorophyll a, total chlorophyll, and carotenoid contents, which means it provided an adequate photosynthetic material basis for the efficient growth of seedlings. Red light (L7) and blue light (L3) favored the accumulation of soluble sugars, while blue light (L3) significantly promoted the synthesis of soluble proteins and proline. In contrast, green light (L5) induced severe oxidative stress, resulting in a significant increase in malondialdehyde content, accompanied by an abnormal increase in the activity of antioxidant enzymes, which inhibited the growth of histocultured seedlings. The comprehensive evaluation results, derived from principal component analysis and the affiliation function method, showed that the adaptability of *A. sinensis* seedlings to different light qualities was ranked as follows: blue-green light (L4) > blue light (L3) > red light (L7) > white light (CK) > blue-violet light (L2) > orange light (L6) > green light (L5) > purple light (L1). Among them, blue-green light (L4), blue light (L3), and red light (L7) all had greater adaptability than that of the normal white light control, and they were the light qualities most suitable for the proliferation of *A. sinensis* seedlings; however, the adaptability of purple light (L1) also stood out, as purple light (L1) was the least adaptable. The observed growth and physiological differences under various light qualities are likely the result of combined regulation by both spectral composition and photosynthetic photon flux density (PPFD). Future optimization of lighting regimens should therefore take both factors into consideration. This study provides an important theoretical basis and practical guidance for the precise light environment regulation in the production of *A. sinensis* group culture plants.

## Figures and Tables

**Table 1:** Effects of different LED (light-emitting diode) light quality on the proliferation and growth of *Aquilaria sinensis* tissue-cultured seedlings.

Light Quality Treatments	Multiplication rate (Number)	Plant Height (cm)	Shoot Growth
CK	3.13 ± 0.09 c	2.49 ± 0.03 b	Leaf blades light green, good length, small amount of healing tissue at base
L1	2.1 ± 0.09 e	2.08 ± 0.03 c	Leaf blades intensely green, poorly growing, with more healing tissue at the base
L2	2.03 ± 0.11 e	1.94 ± 0.01 d	Leaf blades yellowish, poorly grown, with small amounts of healing tissue at base
L3	3.53 ± 0.09 b	2.52 ± 0.02 b	Leaf blades light green, good growth, few healing groups at base
L4	4.07 ± 0.13 a	2.57 ± 0.04 ab	Leaf blade intensely green, good growth, small amount of healing tissue at base
L5	2.07 ± 0.13 e	1.63 ± 0.02 e	Leaf blade light green, poor growth, more healing tissue at base
L6	2.77 ± 0.14 d	1.53 ± 0.02 f	Leaf blade light green, poor growth, more healing tissue at base
L7	4.1 ± 0.13 a	2.63 ± 0.03 a	Leaf blade intensely green, good growth, small amount of healing tissue at base

The data in the table are presented as mean ± standard error. Different lowercase letters indicate significant differences between treatments (*p* < 0.05). CK: white light; L1: purple light; L2: blue-violet light; L3: blue light; L4: blue-green light; L5: green light; L6: orange light; L7: red light. The photosynthetic photon flux density (PPFD) for CK, L1, L2, L3, L4, L5, L6, and L7 was 39.084, 27.451, 85.620, 142.324, 130.849, 73.351, 27.688, and 64.056 µmol m^−2^ s^−1^, respectively.

**Table 2:** Matrix of component score loadings.

Index	Principal Component Coefficient			
	C1	C2	C3	C4
Value-added coefficient	0.138	0.14	−0.133	0.055
Plant height	0.114	0.189	−0.022	0.236
Chlorophyll a	0.143	−0.074	0.097	0.38
Chlorophyll b	0.143	0.02	0.177	−0.289
Total chlorophyll	0.152	−0.049	0.128	0.196
Carotenoids	0.143	−0.088	0.104	0.327
Soluble sugar	0.055	0.328	−0.148	−0.118
Soluble protein	0.097	−0.184	0.111	−0.483
Proline	0.086	0.125	0.258	−0.36
Malondialdehyde	−0.073	−0.244	0.22	0.052
Superoxide dismutase (SOD)	−0.089	0.289	0.115	−0.005
Peroxidase (POD)	−0.102	0.108	0.325	0.402
Catalase (CAT)	0.039	−0.140	−0.454	0.100
Characteristic root	6.195	2.484	1.787	1.017
Contribution rate%	47.652	19.11	13.749	7.826
Cumulative contribution%	47.652	66.762	80.511	88.338

**Table 3:** Comprehensive evaluation of the adaptability of different light qualities in *Aquilaria sinensis* tissue-cultured seedlings.

Light Quality Treatments	Principal Component Score				Value of the Affiliation Function				Consolidated Assessed Value	Rankings
	C1	C2	C3	C4	C1	C2	C3	C4		
CK	0.67	0.49	−1.24	−0.42	0.74	0.65	0.11	0.45	0.53	4
L1	−0.51	−1.23	−1.56	−0.44	0.35	0.00	0.00	0.45	0.20	8
L2	0.12	−0.75	1.25	0.11	0.56	0.18	1.00	0.62	0.49	5
L3	0.52	1.30	1.00	−1.87	0.69	0.95	0.91	0.00	0.64	2
L4	1.44	−0.89	0.48	1.07	1.00	0.13	0.72	0.92	0.67	1
L5	−1.53	−0.13	0.51	0.49	0.00	0.41	0.74	0.74	0.24	7
L6	−0.95	−0.22	0.10	−0.26	0.20	0.38	0.59	0.51	0.29	6
L7	0.25	1.43	−0.54	1.32	0.60	1.00	0.36	1.00	0.61	3

CK: white light; L1: purple light; L2: blue-violet light; L3: blue light; L4: blue-green light; L5: green light; L6: orange light; L7: red light. The photosynthetic photon flux density (PPFD) for CK, L1, L2, L3, L4, L5, L6, and L7 was 39.084, 27.451, 85.620, 142.324, 130.849, 73.351, 27.688, and 64.056 µmol m^−2^ s^−1^, respectively.

## Data Availability

The raw data supporting the conclusions of this article will be made available by the authors on request.

## Supplementary Materials

The following supporting information can be downloaded at: https://www.mdpi.com/article/10.3390/life15111770/s1, Table S1: Linear Expressions for Each Principal Component.
