# Supplementary material for: Effects of Different Light Qualities on Proliferation and Physiological Characteristics of Aquilaria sinensis Tissue-Cultured Seedlings
Source: Life (Basel). 2025 Nov 19;15(11):1770. doi: 10.3390/life15111770 (PMC12653676; doi:10.3390/life15111770)
Supplement: Supplementary file 1 [file life-15-01770-s001.zip › life-3961264-supplementary.pdf]

Supplementary Table S1. Linear Expressions for Each Principal Component

**Number**	**linear expressions**
1	$C_1=0.138X_1+0.114X_2+0.143X_3+0.143X_4+0.152X_5+0.143X_6+0.055X_7+0.097X_8+0.086X_9-0.073X_{10}-0.089X_{11}-0.102X_{12}+0.039X_{13}$
2	$C_2=0.140X_1+0.189X_2-0.074X_3+0.020X_4-0.049X_5-0.088X_6+0.328X_7-0.184X_8+0.125X_9-0.244X_{10}+0.289X_{11}+0.108X_{12}-0.140X_{13}$
3	$C_3=-0.133X_1-0.022X_2+0.097X_3+0.177X_4+0.128X_5+0.104X_6-0.148X_7+0.111X_8+0.258X_9+0.220X_{10}+0.115X_{11}+0.325X_{12}-0.454X_{13}$
4	$C_4=0.055X_1+0.236X_2+0.380X_3-0.289X_4+0.196X_5+0.327X_6-0.118X_7-0.483X_8-0.360X_9+0.052X_{10}-0.005X_{11}+0.402X_{12}+0.100X_{13}$
